# Orientation-dependent photonic bandgaps in gold-dust weevil scales and their titania bioreplicates

**DOI:** 10.3762/bjnano.16.1

**Published:** 2025-01-02

**Authors:** Norma Salvadores Farran, Limin Wang, Primoz Pirih, Bodo D Wilts

**Affiliations:** 1 Department for Chemistry and Physics of Materials, University of Salzburg, Jakob-Haringer-Str. 2a, 5020 Salzburg, Austria; 2 Current address: Institute of Materials Science and Technology, Vienna Technical University, 1060 Vienna, Austria

**Keywords:** animal coloration, photonic crystal, polarization conversion, sol–gel replication, weevil

## Abstract

The scales of the gold-dust weevil *Hypomeces squamosus* are green because of three-dimensional diamond-type chitin–air photonic crystals with an average periodicity of about 430 nm and a chitin fill fraction of about 0.44. A single scale usually contains one to three crystallites with different lattice orientations. The reciprocal space images and reflection spectra obtained from single domains indicated a partial photonic bandgap in the wavelength range from 450 to 650 nm. Light reflected from {111}-oriented domains is green-yellow. Light reflected from blue, {100}-oriented domains exhibits polarization conversion, rotating the angle of linearly polarized light. The overall coloration, resulting from the reflections from many scales, is close to uniformly diffuse because of the random orientation of the domains. Using titania sol–gel chemistry, we produced negative replicas that exhibited a 70 to 120 nm redshift of the bandgap, depending on the lattice orientation. The wavelength shift in {100} orientation is supported by full-wave optical modeling of a dual diamond network with an exchanged fill fraction (0.56) of the material with the refractive index in the range of 1.55 to 2.00. The study suggests that the effective refractive index of titania in the 3D lattice is similar to that in sol–gel films. The study demonstrates the potential of replicating complex biophotonic structures using the sol–gel technique. Optimization of the sol–gel process could lead to customizable photonic bandgaps that might be used in novel optical materials.

## Introduction

Animal coloration is produced by means of absorption, scattering, luminescence, and interference. The latter is achieved when light interacts with a material having a periodically changing refractive index. Interference produces structural colors that may be quite saturated and angle-dependent (iridescent). Structurally colored materials feature refractive index variations in one, two, or three dimensions [1–2]. In particular, three-dimensional (3D) photonic crystals are characterized by a photonic bandgap that prohibits light of certain wavelengths from propagating through (specific) orientations of the material [3]. A complete photonic bandgap, where propagation of light waves in a certain wavelength band is prohibited, is generally not possible with biological photonic crystals, as the refractive index contrast (approx. 1.55) between chitin and air is too low to achieve this condition [4–5].

The intriguing properties of photonic bandgap materials extend beyond coloration, as they can be used for manipulating light in key optical technologies such as lasers [6], light-emitting diodes [7], and light guides [8]. For applications in the visible spectral range, the periodicity needs to be in the range of a few hundreds of nanometers [9]. This periodicity is challenging to engineer using, for example, block copolymers [10–11], lithography, or laser etching [12–13], but it can be routinely found in animal integuments.

Biomimetic approaches using templates from natural structures offers a possible alternative. The scales of many beetles and weevils contain diamond photonic crystals [14–16] that may serve as a template for materials with a complete photonic bandgap, provided that the refractive index contrast of the template is increased [15,17–18]. Birds, butterflies, beetles, and, particularly, weevils produce many interesting quasi- and highly ordered photonic structures [14,19–22] that may be used as an inspiration for optical engineering. Indeed, single diamond photonic networks are one of the most efficient naturally occurring 3D photonic crystal structures. They can also be described using triply periodic minimal surface (TPMS) models, where a minimal surface separates two volumes with differing refractive indices. The diamond TPMS structure is special as it provides optimal diffraction efficiency and can form photonic bandgaps even with lower refractive index contrasts (i.e., with refractive index contrasts above 2.1) [15,17]. This makes these structures rather interesting for their use as templates. The work of Galusha and colleagues demonstrated a double-imprint templating process to create a positive titania replica of beetle scales with high refractive index. This showed the effectiveness of a sol–gel process at relatively low temperature (130 °C) in replicating complex biological structures and obtaining photonic crystals with desired optical properties [23–24].

In this work, we characterized natural photonic crystals found in the scales of the gold-dust weevil, *Hypomeces squamosus* (Coleoptera: Curculionidae; Herbst 1795), a pest of several crops [25–27]. We investigated the properties of photonic structures in the scales using reflected light microscopy and spectroscopy. Brillouin zones inferred from the reciprocal space images indicated that the scales are composed of one to three randomly oriented domains of single diamond network photonic crystals. The domains in lattice orientation {100} exhibited polarization conversion. The structure inferred from optical measurements was confirmed using conventional and focused ion beam scanning electron microscopy (FIB-SEM). By averaging the reciprocal space images obtained from different lattice orientations, we obtained a uniform diffuse green scatterogram, confirming the mechanism producing the dull, unsaturated overall coloration found on the gold-dust weevil and several other weevils [19,21,28]. Subsequently, using plasma etching, we removed the scale cortex and made negative titania replicas of exposed photonic crystals using a sol–gel chemistry approach [14,24]. The increased refractive index contrast and the increased fill fraction of the replicas resulted in a redshift, which was confirmed with full-wave modeling. Full-wave modeling further suggested that the titania replicated from a 3D template has a similar refractive index as the titania thin layers obtained with a sol–gel process, suggesting a similar porosity despite the more complicated geometry. The work further supports the use of biological photonic structures for synthesizing novel optical devices.

## Results

### Appearance and structure of the scales

**Figure 1:**
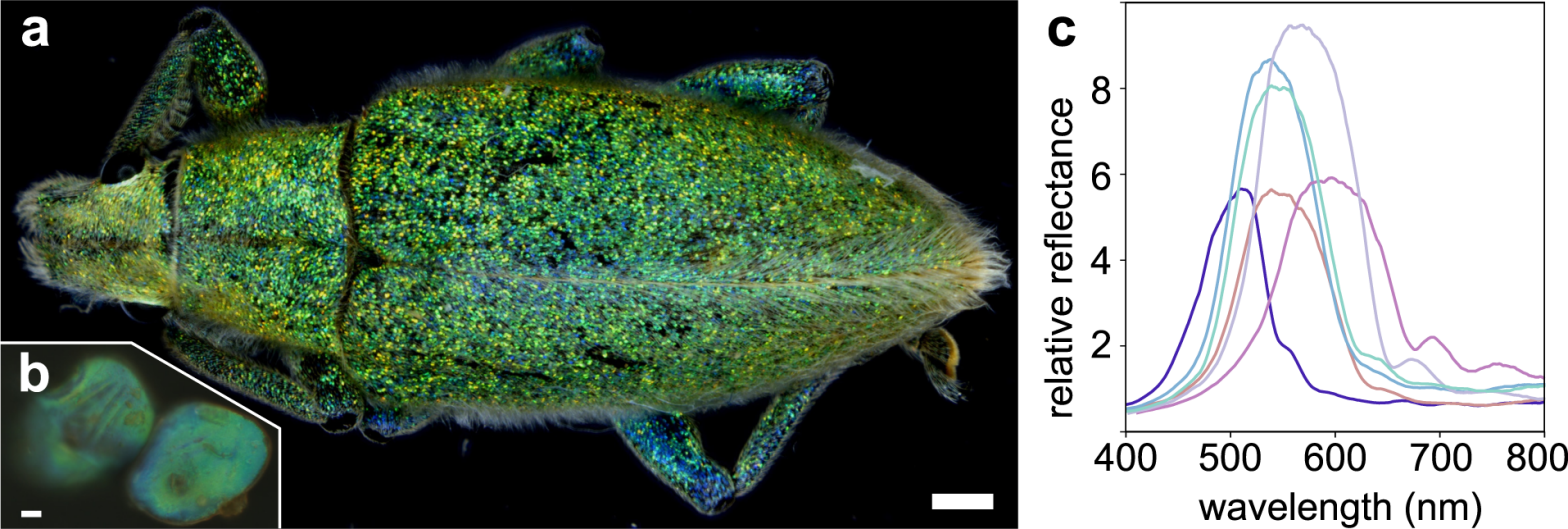
The gold-dust weevil *Hypomeces squamosus*. (a) A macro photo of the weevil with extended depth of field. (b) Epi-reflectance microscope image of two scales, upper (left) and lower side (right) facing upwards (average scale length 67 ± 7 μm, *N* = 10). (c) Reflectance spectra of several single scales, measured with the 20× (NA = 0.60) objective and normalized against a white diffusive standard. Scale bars: (a) 1 mm and (b) 10 μm.

The body and the elytra of the gold-dust weevil *Hypomeces squamosus* are covered with iridescent scales (Figure 1). The scales on the wings and body appear mostly greenish, while the scales on the legs are more bluish (Figure 1a). The elytral scales are flat, about 70 μm long and about 50 μm wide. Several parallel lines are visible on the upper side of the scales at high magnification in an optical microscope, and the broken scale stalks are visible on the lower side (Figure 1b).

**Figure 2:**
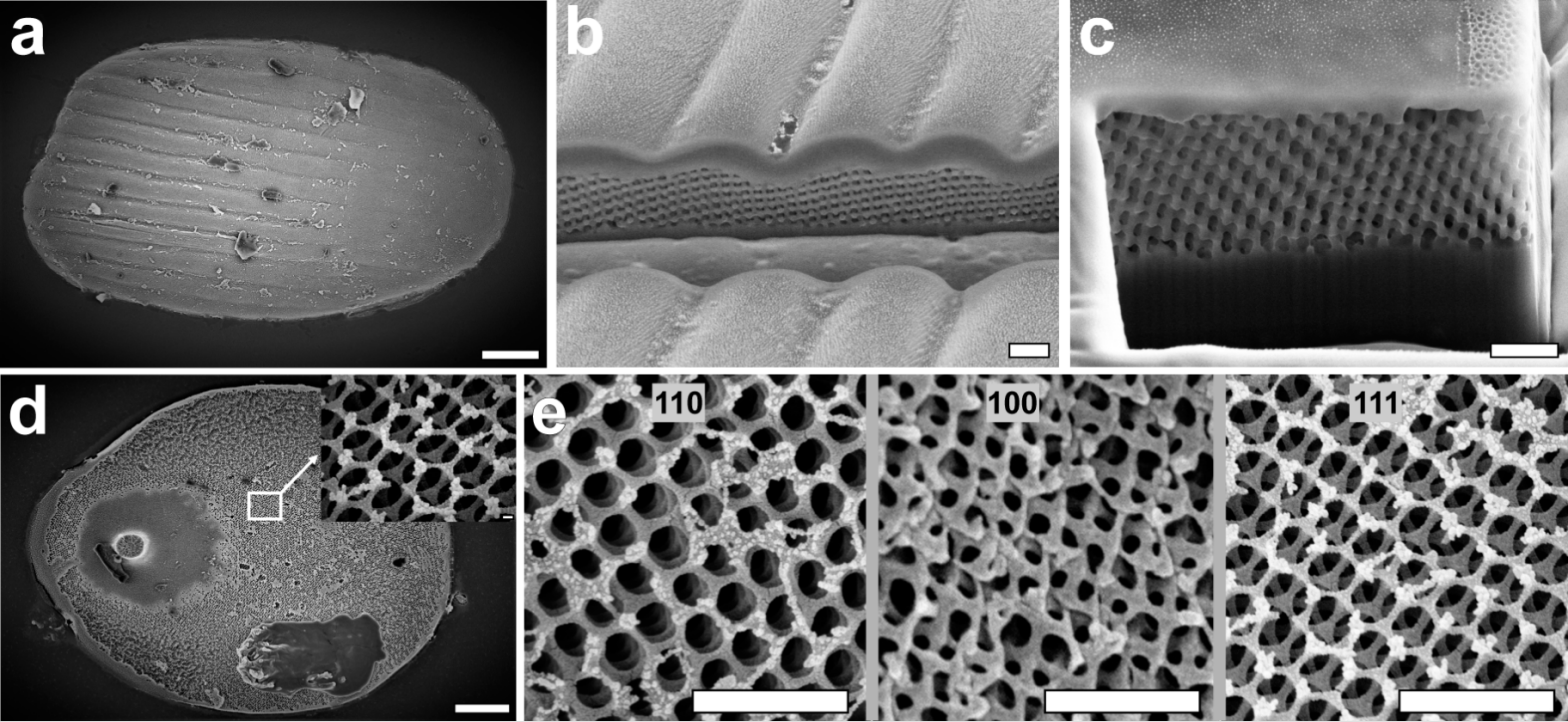
Structural characterization of the scales. (a) SEM image of an intact scale with the upper lamina facing upwards. (b, c) Cross-sectional images of an intact scale show a thin cortex wrapping around a chitin–air network with a 3D periodic nanostructure; the undulating upper cortex layer is thicker in (b) than the flat lower cortex layer in (c). (d) SEM image of the lower surface of an etched scale. (e) Different lattice orientations of the 3D nanostructure found in etched scales. The structure is strongly reminiscent of single diamond-type photonic networks [15]. Scale bars: (a, d) 10 μm, inset 100 nm, (b, c) 1 μm, and (e) 2 μm.

Single scales, imaged with the 20× (NA = 0.60) objective and full-aperture illumination, appear rather uniformly colored (Figure 1b). Reflectance spectra measured with a modified reflected light microscope (see Methods section) feature peaks between 450 and 600 nm (Figure 1c), regardless of which scale side is facing upwards. The reflectance amplitudes are markedly higher than that of a diffuse reference standard, indicating directional reflection. The scales immersed in refractive index oils appeared yellowish because of a presence of a blue-absorbing pigment (Supporting Information File 1, Figure S1). Maximal transmission was achieved with the oil with refractive index 1.56, indicating that the scales are composed of chitin and an undetermined short-wavelength absorbing pigment. To investigate whether the origin of the coloration is structural, we examined the external and internal structure of intact and plasma-etched elytral scales using scanning electron microscopy (Figure 2). Using a focused ion beam electron microscope, we exposed the diamond lattice chitinous network underneath the cortex of intact scales (Figure 2b,c). The upper cortex is ≈1 μm thick and has undulations spaced about 5 μm apart (Figure 2b), which were visible in the light microscopy images ( Figure 1b). The lower cortex is thinner (≈0.5 μm) and flat (Figure 2c).

From the FIB-SEM cuts, we estimated the chitin fill fraction of the chitin network to be 0.44 ± 0.06. By adjusting the power and duration of the argon plasma etching, we were able to selectively etch the lower cortex of the scales (Figure 2d), revealing the underlying chitin network (Figure 2d,e). The etched scales showed different lattice domains that had a reduced chitin fill fraction due to etching. From the 2D Fourier transform of the {111}-oriented domains, whose first spatial frequency peaks were in a symmetric hexagonal pattern, we estimated a single-diamond unit cell size of 427 ± 4 nm (Supporting Information File 1, Figure S3). The transition between differently oriented domains is continuous (Supporting Information File 1, Figure S5).

### Spatial distribution of reflectance

**Figure 3:**
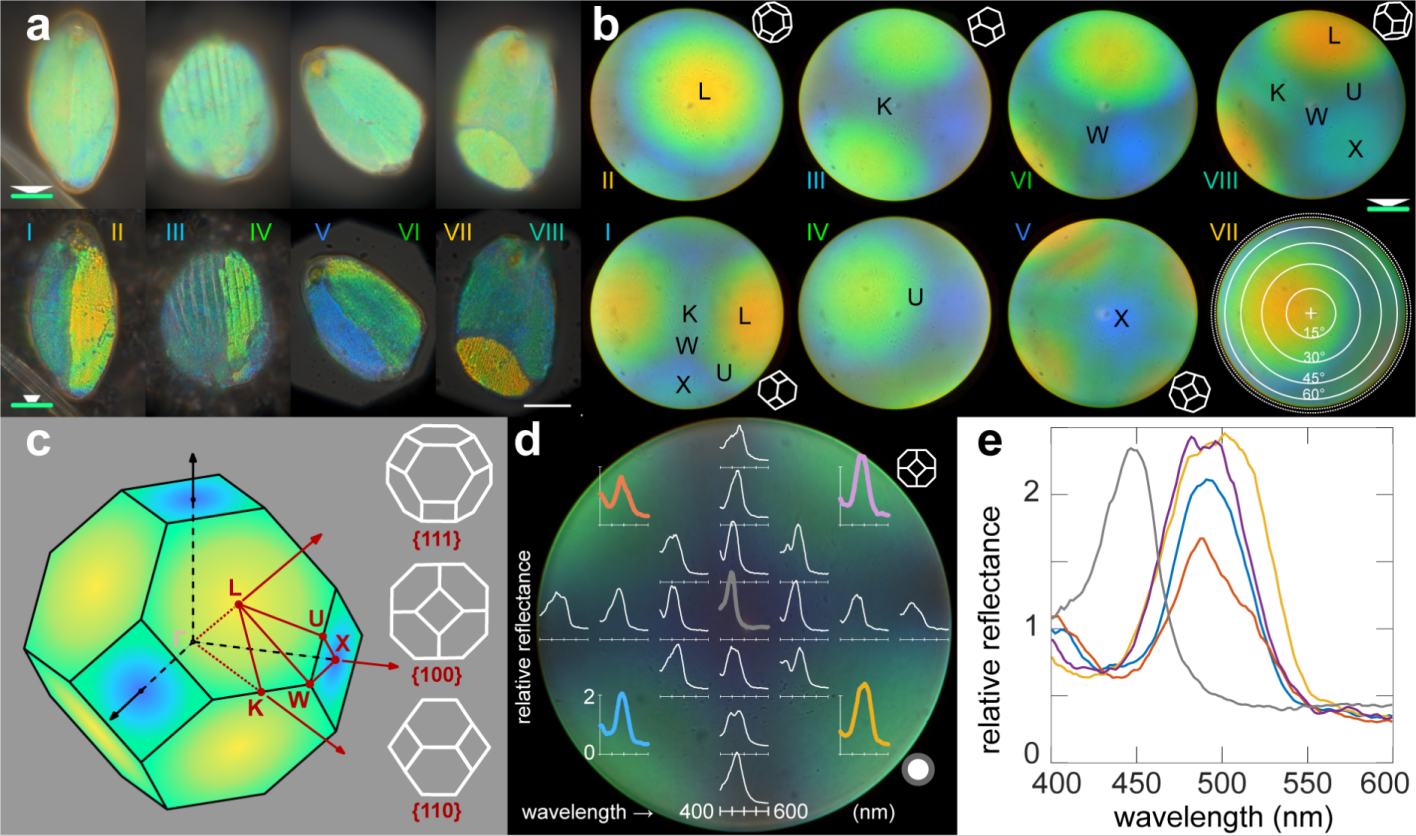
Real and reciprocal space images and directional reflectance spectra. (a) Light microscopy images of four scales, each with two domains, illuminated with large-aperture (top row) and with small-aperture (bottom row) illumination, revealing the differently colored domains. (b) Reciprocal space images obtained by illuminating the domains shown in (a) with small-field, full-aperture illumination reveal the first Brillouin zone with indicated symmetry points. See Supporting Information File 1, Figure S6, for more examples. (c) A schematic representation of the first Brillouin zone of a diamond network (left) and the isometric projections of the high-symmetry points (right). (d, e) Directional reflectance spectra measured from the reciprocal image of a {100}-oriented domain. Polarization properties of the same domain are shown in Supporting Information File 1, Figure S2. Reflectance spectra were normalized against a white diffusive standard. Roman numerals indicate the domains corresponding between (a) and (b). Scale bar: (a) 20 μm. The rim of the reciprocal space images (b, d) corresponds to 71° (NA = 0.95). Symmetry points in (b, c) are indicated with letters (LUXWK). The white circle in (d) approximates the collection area of the *XY*-translatable fiber.

Previous work on single diamond network photonic crystals has shown that their orientation and nature can be identified using reciprocal *k*-space imaging [28]. To see whether the gold-dust weevil scales contain diamond photonic crystal networks, we performed reciprocal space imaging using a 50× (NA = 0.95) objective. The real space images with full-aperture illumination have shown a uniform green tint of the scales. With the illumination aperture fully closed, the real space images revealed domains with distinct blueish, greenish, and yellowish tints (Figure 3a).

By illuminating a single domain with full-aperture illumination and inserting the Bertrand lens into the detection pathway, we obtained reciprocal space images (Figure 3b). The domains oriented close to the {111} direction produced reciprocal space images with a greenish or yellowish central hexagon shape (Figure 3b II,VII), while the domains close to {110} orientation produced images with two partially visible hexagons (Figure 3b I,III,VIII). The domains close to the {100} orientation exhibited a square-shaped blue zone in the center of the reciprocal space image, corresponding to the X-symmetry point (Figure 3b V). Overall, the domains in the scales seem to be oriented randomly. Additional measurements are presented in Supporting Information File 1, Figure S6.

Reciprocal space imaging also allows one to map the bandgap diagram. For this, we measured reflectance spectra in the reciprocal space using full-aperture illumination from a domain that was almost perfectly {100}-oriented (Figure 3d). The spectrum measured in the image center, close to the X-symmetry point, has a peak around 440 nm, whereas the spectra measured at the diagonals close to the rim, approaching L-symmetry points, have peaks around 500 nm (Figure 3e). The reflectance peak from the L-symmetry point, measured from a {111}-oriented domain, was about 550 nm (not shown here). The polarization dependence of reflections is shown in Supporting Information File 1, Figure S2. In brief, while the {111}-oriented domains retained the polarization angle, the light reflected from {100}-oriented domains exhibited polarization conversion, similarly to the case shown in [16]. The polarization contrast (i.e., the ratio of maximal to minimal peak reflectance) was lower for the blue domain than for the green domain. The polarization conversion angle depended on the exact orientation of the domain.

### Titania replicas exhibit a redshift

Diamond-type photonic crystals are arguably one of the most sophisticated structures of photonics research [14,28–29], but the refractive index of chitin (*n* = 1.55, see also [4]) in intact insect scales is too low to allow for the formation of a complete bandgap, hindering photonic applications. The diamond networks in insect scales are, however, ideal templates for transferring these complex geometries, which cannot be otherwise synthesized, into media with higher refractive index. Plasma etching is an effective way to open the beetle scales and make them accessible to the chemical environment used for biotemplating. To show the potential of the gold-dust weevil scales for biotemplating, we plasma-etched the scales and produced (imperfect) negative replicas of the diamond-based photonic structure via an established titania-based sol–gel process [24], followed by the removal of the chitinous template. The elemental composition of the original scales and the replicas (Supporting Information File 1, Table S1) shows that nitrogen is absent in the replicas, indicating that the cleaning procedure, despite its relatively low maximal temperature (130 °C) satisfactorily removed the template’s original material from the replica.

**Figure 4:**
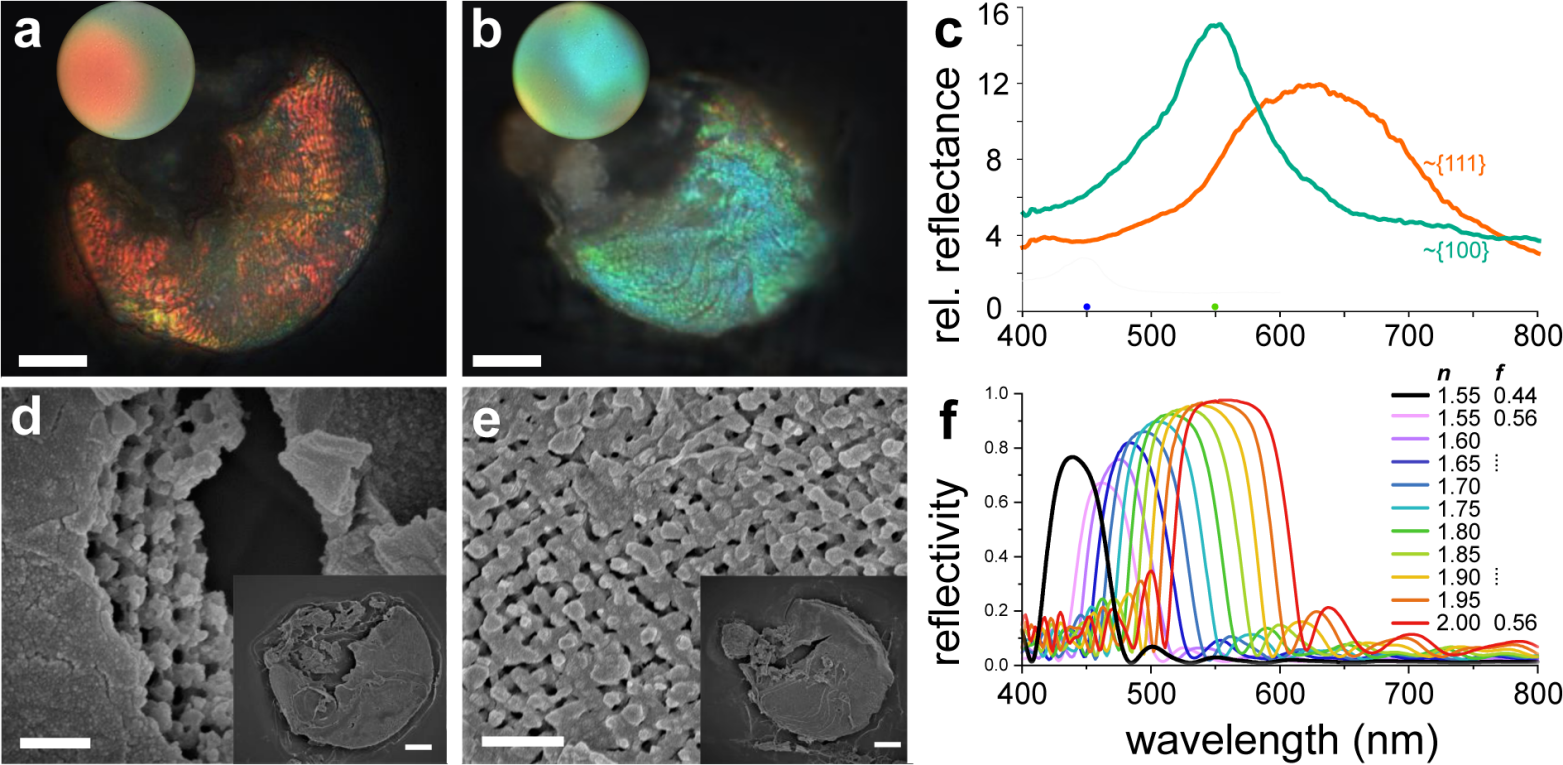
Structural properties of negative titania diamond scale replicas, optical measurements, and optical modeling. (a, b) Real space microscopy images, the insets show the associated reciprocal space images. (c) Reflectance spectra of the replicas. (d, e) High-magnification SEM images, obtained from the same replicas as shown in panels (a, b). (f) Full-wave optical modeling of the {100} orientation of a single diamond network photonic crystal with refractive index *n* = 1.55 and fill fraction *f* = 0.44, and the negative replica with fill fraction *f* = 0.56, in the refractive index range *n* = 1.55–2.00. Scale bars: (a, b) 10 μm, (d, e) 1 μm (in the insets 10 μm). The rim of the reciprocal space image in the insets of (a, b) corresponds to 71° (NA = 0.95).

We obtained two different types of replicas differing in color; the first type was orange, and the second type was greenish-blue, as shown in Figure 4. The real space and reciprocal space images are shown in Figure 4a,b. The green replica was roughly {100}-oriented, and the orange replica was roughly {111}-oriented. SEM images shown in Figure 4d,e confirmed the successful replication with an increased fill fraction, compared to the original material (Figure 2); the two fill fractions, *f*, were expected to be conserved but exchanged (i.e., titania *f* ≈ 0.56 and void *f* ≈ 0.44).

The obtained reflectance spectra of the green and the orange replica (Figure 4e) have peaks and bandwidths (FWHM) of 550 ± 50 and 625 ± 150 nm, respectively. The comparison with the spectra of the original scales with similar domain orientations (blue 440 nm, green 550 nm) indicate a redshift of the peak reflectance of about 110 and 125 nm, for the {100}- and {111}-oriented domains, respectively. The spectra of replicated domains that were less ideally oriented are shown in Supporting Information File 1, Figure S4. The redshift is consistent both with a higher refractive index contrast and with an increase of the fill fraction.

### Modeling confirms an increase of refractive index contrast

Full-wave simulations of a {100}-oriented diamond photonic crystal with a range of refractive indices (Figure 4f) show that the reflectivity (i.e., the angle-integrated reflectance) of the idealized diamond structure with fill fraction *f* = 0.44, illuminated with a plane wave, peaks at about 440 nm, and that a shift to 540 nm is expected for a negative replica (*f* = 0.56) at the refractive index *n* = 1.90, with a concomitant approx. 25% increase of peak reflectivity (from ≈0.75 to ≈0.95). The increase of reflectivity shown by modeling is consistent with the measured peak reflectance in original and templated scales (original: <10, template: >10, referred to a diffuse white standard (Figure 1 and Figure 4c). The measured spectra, both of the native templates and the replica, are broader than those produced by the idealized model.

## Discussion

The scales are not just a simple covering for the gold-dust weevil; they are, in fact, a complex photonic system. The green hue of the scales arises from the interaction of light with the three-dimensional diamond-type chitin–air photonic crystal structures present within the scales (Figures 1–3). This diamond network, with a unit cell constant of approximately 430 nm and a chitin fill fraction of slightly less than a half, interacts with incident light in a way that results in angle-dependent coloration and polarization on a microscopic level (Figure 3 and Supporting Information File 1, Figure S2). This phenomenon is similar to what has been previously observed in other weevils that possess diamond-type photonic crystals [14–15,30] with similar chitin filling fractions [31–32] and unit cell dimensions [15]. The elytral scales are not uniform, as they can contain single or multiple crystallites with varying lattice orientations under a structured enveloping cortex. Each crystallite contributes to the overall optical properties of the scale (Figure 3). The crystallites can feature different orientations, which has strong consequences for interference and, thus, the resulting color [3,5].

The domains oriented roughly along the {100} direction exhibit polarization conversion (Supporting Information File 1, Figure S2 and [16]). This property is indeed interesting from a materials science perspective, but it is unlikely that polarization conversion would give an adaptive advantage in biological signaling, as the polarization signal is scrambled by multiple crystallite orientations and across the scales. Indeed, we observed virtually no noticeable effect on iridescence when using a polarizer and/or analyzer at the macrophotographic scale; however, a subtle iridescent effect on the elytral scales was observed in a particular experimental setting, using a (small aperture) stereomicroscope, either with a ring illuminator or an arbitrarily placed point source. With the ring illuminator, the scales facing the objective appeared predominantly blue-green speckled, while, among the scales seen at grazing angles, less scales appeared blue and more turned yellowish (subtly seen in Figure 1a, more pronounced in Supporting Information File 1, Figure S7). A pointillist macroscopic coloration effect by a changing periodicity has been shown for cuticular multilayers [33], but to our knowledge never conclusively for photonic crystals (for a review, see [21]). The iridescence effect was reversed when we turned the animal to the side (not shown); hence, the effect is likely not due to the material composition or lattice periodicity differences between the scales at the top and the side of the elytrae.

Given the randomness of the lattice orientations in the scales of the gold-dust weevil, the iridescence observed in slanted scales is possibly due to the photonic crystals’ finite dimensions and due to the optical effects caused by the enveloping cortex. Moreover, under an extended illumination source, iridescence vanished, and the weevil appeared uniformly diffuse bright green and dull (i.e., without specularity). We simulated the effect of color mixing by averaging the narrow- and whole-aperture-illuminated reciprocal space images and obtained a uniform green far-field reflectance profile (Supporting Information File 1, Figure S7), as shown experimentally previously [15,19]. We, hence, conclude that under natural illumination, the weevil’s intricate photonic effects average out into a uniform green. Diffuse coloration is not uncommon in nature and is achieved either by quasi-ordered and disordered structures [19–20], by employing pigments [34], by randomization of orientations of photonic structures [21,35], or by a combination of these mechanisms [36]. It seems that the additive pointillistic mixing of ordered domains observed in the gold-dust weevil and other weevils is quite good at reducing the specular surface reflections, especially when compared with multilayered cuticles [21].

We employed biotemplating to create negative replicas of weevil scales using well-established titania sol–gel chemistry [37]. These replicas, while maintaining the intricate structure of the original scales, exhibited a redshift of the photonic bandgap of 70 to 120 nm, depending on the orientation (Figure 4). This peak shift can be attributed to the difference in refractive index between chitin, the primary component of the original scales, and titania, the material used for the replica, and the increase of the fill fraction.

The measured reflectance spectra, both of the native templates and the replicas, are broader than those produced by the idealized model. This difference is partially attributed to the measurement geometry: While the model assumes a plane wave, in the experiment, the source is extended, and the light collection is either having a large but finite angle in real space, or a small angle in the reciprocal space. Further, in the experiment, the lattice orientation is never exactly in the {100} orientation.

Taking into account the general arrangement of the investigated material, many factors (e.g., local periodicity imperfections, optical crosstalk between crystallites, and optical effects of the lamina, or, in the case of replicas, depth-dependent etching) may additionally scramble the measured spectra. Comparing the experiments and the model, we therefore suggest that the employed templating procedure produced a titania photonic crystal with an effective refractive index likely in the range of 1.75–1.90 (Figure 4). This is, to our knowledge, the first rough estimation of the refractive index for titania in a 3D lattice, which indicates that with the templating process employed here, the 3D geometry did not significantly increase the porosity of titania.

Our estimate for the effective refractive index of titania in the 3D lattice is in the range reported for pure titania thin films (1.95–2.55 [38], 1.72–2.03 [39]), hybrid silica/titania thin films (1.95 [40], 1.50–1.95 [41] and hybrid organotitania (1.75–2.05 [42], 1.55–1.65 [43]). This is lower than the tabulated values of titania prepared by, for example, layer deposition (2.10 [44], 2.40 [45], see also [46–47]), but higher than that of pure silica replicas (1.50 [18]). This is not unexpected as the effective refractive index of titania thin films produced by sol–gel synthesis varies because of porosity, depending on the specific process, chemicals, and reaction conditions [41]. A higher annealing temperature seems to have a large influence on reducing the porosity of thin films, while, during templating, it unfortunately introduces lattice breakage and shrinkage. Therefore, a calcination process at high temperatures is likely not the best option for pure titania [24], although it seems to work better for silica [18]. The replication process used here had a modestly high temperature step (130 °C). Raising the temperature slightly might increase the effective refractive index of titania before lattice degradation occurs. If the process could be additionally tuned by, for example, varying process times and chemical compositions (including hybrid sol/gels), a further reduction of porosity might lead to the closing of the photonic bandgap at the effective refractive index of about 2.1 [17].

## Conclusion

The gold-dust weevil’s scales house a complex photonic system that influences coloration and light interaction through a three-dimensional diamond-type chitin–air photonic crystal structure. While the individual scales appear bright and of saturated color, the resulting overall coloration under an extended light source is uniformly green with a very low specularity. The synthesis of negative replicas using titania sol–gel chemistry demonstrates its potential for producing materials with length scales found in nature. The observed redshift in the replicas indicates the possibility of tuning optical properties by varying the replication material. Optimizing the existing sol–gel procedures using, for example, (i) a double inversion process, (ii) other materials that would produce a higher refractive index contrast, or (iii) materials with non-linear optical properties [48–50] would be an interesting future research direction.

## Experimental

### Light microscopy

The image of the whole animal was taken with a stereomicroscope (Stereo Discovery V20, Zeiss, Oberkochen, Germany). For microscopy and spectroscopy, we used a Zeiss Axioscope 5 with an epi-illumination attachment equipped with the field and aperture stops and a linear polarizer (0–90°). The observation path included a Bertand lens and a fully rotatable linear analyzer. The objectives used were from the EC Epiplan-Neofluar series [5×, 10×, 20×, 50×, and 100×] with apertures [NA = 0.13, 0.30, 0.60, 0.95, and 0.95], respectively. For reciprocal space imaging, we used the 50× and 100× objectives. The 100× objective has a smaller minimal illumination spot (field), but a bigger minimal aperture (angle of the illumination cone), and produces a smaller reciprocal image size than the 50× objective. We, therefore, used the latter on single-domain scales and with small illumination apertures (Supporting Information File 1, Figure S7), and the former for reciprocal space spectroscopy (Figure 3, Supporting Information File 1, Figure S2). Images were acquired with a RGB camera (DFK 38UX304, pixel pitch 3.45 μm, The Imaging Source, Bremen, Germany) and processed with ImageJ/Fiji [51]. Extended depth-of-field images (Figure 1, Supporting Information File 1, Figure S7) were processed from image stacks with Helicon Focus (HeliconSoft, Kharkiv UA).

### Spectroscopy

Reflectance spectra were collected via a side port (part 425146-9031, Zeiss) installed below the standard tube lens. The side port was modified to fit a 30 mm cage system holding an *XY* translator (ST1XY-S/M, Thorlabs, 6 mm travel) holding a light fiber at the focal plane of the side port’s tube lens. The light was reflected to the side via a slide-in mirror and focused by the tube lens on a 200 μm fiber (FC-UVIR200, Avantes, Apeldoorn NL EU) attached to a CCD spectrometer (AvaSpec-ULS2048XL-EVO, Avantes). The measurement spot could be translated using the micrometer screws, whose readings were calibrated using reverse illumination and a mirror in the object plane. For spectroscopy of reciprocal space images (Figure 3, Supporting Information File 1, Figure S2), we used the 100× (NA = 0.95) objective. For spectroscopy of real space images (Figure 1), we used the 20× (NA = 0.60) objective. A white diffuse reference tile (WS-2, Avantes) was used as the reference for spectroscopy measurements. Illumination was provided by a xenon or a halogen light source.

### Electron microscopy

Top-view images of single scales were acquired with a Zeiss Ultra Plus 55 scanning electron microscope (Zeiss, Oberkochen, Germany) using an in-lens secondary electron detector at 5 kV acceleration voltage. The scales and replicas were attached to an aluminum holder via a conductive carbon tape. These samples were coated with gold using a Sputter Coater 108 (Cressington Scientific Instruments, Watford, UK). The settings were: sputter time 120 s, current 40 mA, and background pressure 0.08 mbar. Cross-sectional images of single scales were recorded using a focused ion beam scanning electron microscope (Scios2, Thermo Fisher Scientific, Waltham, MA, USA) using an Everhart–Thornley detector and an in-lens secondary electron detector at 5 kV acceleration voltage. These samples were coated with gold using a Cressington 208 HR sputter coater. Elemental analysis was performed with 8 kV acceleration voltage, using an EDX detector attached to the Zeiss SEM (X-Max silicon drift energy-dispersive spectrometer, detector area 50 mm^2^, Oxford Instruments, UK).

### Lattice periodicity

The lattice periodicity was estimated from SEM images using ImageJ/Fiji [51]. Briefly, SEM images were calibrated using the annotated scale bar and converted from 8-bit to float. Domains with a consistent hexagon appearance were cropped to a square and windowed using a circular Gaussian-smoothed mask. We analyzed images whose 2D Fourier transforms had six symmetric and sharp amplitude peaks. The periodicity *k* of the orientation {111} was estimated as the average of the distances of the three unique peaks from the DC point, and the nearest neighbor distance 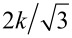 was multiplied with 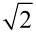 to obtain the unit cell size.

### Preparation of titania replicas

Individual scales were scraped off from the elytra and transferred to a glass slide. Scales were etched using an argon plasma etching device (Emitech K1050X, Quorum, Laughton, UK). The settings were: etching time 36 min, power 50 W, argon pressure 0.9 mbar. Negative titania replicas were fabricated from the etched scales using a sol–gel chemistry protocol modified from ([24]). Briefly, titania sol was synthesized by adding 2 mL of hydrolyzed titanium ethoxide to a pre-mixed solution containing 1.6 mL of concentrated trifluoroacetic acid (99%) and 0.4 mL of concentrated hydrocloric acid (12 M). After stirring the mixture for 20 min, 4 mL of ethanol was added to adjust the viscosity of the solution. Titania sol was formed by stirring the mixture continuously for 24 h. Two drops of 1 μL of titania sol were dripped onto the etched weevil scales at opposing points and a glass slide was placed on top to ensure even infiltration via capillary forces. To solidify the sol and evaporate the residual solvent, the scales were heated in an oven at 100 °C for 20 min. Then, the top slide was removed. The scale template was removed by acid etching, using drops of a 3:1 mixture of concentrated nitric and hydrochloric acids, followed by heating at 130 °C for 15 min. The replicas were then cleaned using deionized water. Titanium(IV) ethanolate (33–35% TiO_2_), tetraethyl orthosilicate (TEOS 98%), and the standard chemicals (trifluoroacetic acid, nitric acid (68%, technical), hydrochloric acid (35%, technical), and perchloric acid (60%)) were purchased from VWR (Vienna, Austria).

### Full-wave photonic modeling

Light scattering by the single diamond network nanostructure was simulated with the three-dimensional finite-difference time-domain (FDTD) method, using Ansys Lumerical 2024 R1 (Ansys Inc., Canonsburg, PA, USA). The diamond nanostructures were approximated via an idealized single diamond network approximated by triply periodic minimal surface model from its level-set equation [52]. Diamond geometries were set up in a rectangular simulation box with two lateral directions. While the in-plane boundaries had periodic boundary conditions, the boundary along the incident light directions had a perfectly matched layer (PML) boundary. The diamond geometry used to simulate the scale response had a lattice parameter of 400 nm and a solid fill fraction of 0.44, with a refractive index of cuticular chitin [4]. Simulations of the negative template had the inverse network with a solid fill fraction of 0.56 and a refractive index between 1.55 and 2.00. Light, with wavelengths of 400–800 nm, was incident in normal direction onto the structure that was oriented along the [100] direction. The angle-integrated reflectivity was obtained from a monitor placed above the light source spanning the entire simulation box area.

## Supporting Information

File 1 Material characterization, polarization properties, lattice estimation, additional microscopy and spectroscopy.

## Data Availability

The data that supports the findings of this study is available from the corresponding author upon reasonable request.
