# Supplementary material for: Orientation-dependent photonic bandgaps in gold-dust weevil scales and their titania bioreplicates
Source: Beilstein J Nanotechnol. 2025 Jan 2;16:1–10. doi: 10.3762/bjnano.16.1 (PMC11702294; doi:10.3762/bjnano.16.1)
Supplement: File 1 — Material characterization, polarization properties, lattice estimation, additional microscopy and spectroscopy. [file Beilstein_J_Nanotechnol-16-01-s001.pdf]

BEILSTEIN JOURNAL OF NANOTECHNOLOGY

# Supporting Information

for

# Orientation-dependent photonic bandgaps in gold-dust weevil scales and their titania bioreplicates

Norma Salvadores Farran, Limin Wang, Primoz Pirih and Bodo D. Wilts

*Beilstein J. Nanotechnol.* **2025,** *16,* 1–10. doi:10.3762/bjnano.16.1

## Material characterization, polarization properties, lattice estimation, additional microscopy and spectroscopy

License and Terms: This is a supporting information file under the terms of the Creative Commons Attribution License (https://creativecommons.org/licenses/by/4.0). Please note that the reuse, redistribution and reproduction in particular requires that the author(s) and source are credited and that individual graphics may be subject to special legal provisions.
The license is subject to the *Beilstein Journal of Nanotechnology* terms and conditions: (https://www.beilstein-journals.org/bjnano/terms)

## Characterization of the scale refractive index

Individual scales were deposited on a glass slide and immersed in oils of known refractive index (Cargille Refractive index Liquids: Catalog 18005, Set: RF 1/5). Transmission spectra were taken using a spectrometer coupled to a visible light microscope. When the refractive index matches that of the scale material, the scattering is minimized and the measured transmittance is maximal. We have immersed the scales into five immersion oils with refractive indices in the range $[1.52 : 0.02 : 1.60]$. For each oil, 20 spectra were taken and averaged (Figure S1.) A decreased transmission below 550 nm is due to chitin and likely due to another short-wavelength (yellow) absorbing pigment. The transmittance in the range $[650 : 1000\,\mathrm{nm}]$ where there is no absorption or possible bandgap effects, was maximal with the oil with refractive index 1.56, in accordance with the refractive index value reported for chitin ([1]).

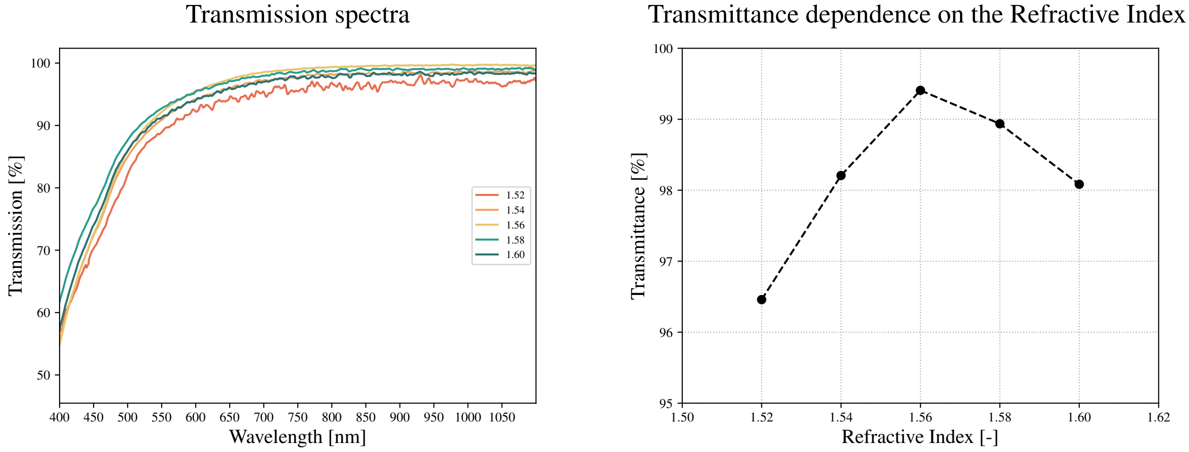


Figure S1: (a) Transmittance spectra of single scales immersed in different refractive index oils $[1.52, 1.54, 1.56, 1.58, 1.60]$, measured with $100\times$ (NA = 0.95) objective. (b) Average transmittance in the wavelength range as a function of oil refractive index $[650 : 1000\,\mathrm{nm}]$

## Polarization conversion in blue $\{100\}$-oriented domains

We investigated whether the differently oriented domains convert polarization, as previously shown by Wu and colleagues [2]. For this, we inserted a linear polarizer into the illumination path and noticed that the real space images of blue and green domains were brightest at different analyzer angles (Fig. S2a). The light reflected from green $\{111\}$-oriented domains retained the polarization

angle, while in this case, the light reflected from the {100}-oriented blue domains had the polarization angle rotated for about 50°(Fig. S2b). The same phenomenon was observed for spot-illuminated single domains in the reciprocal space (Fig. S2c,d). The reflectance spectra measured close to the symmetry points had the peaks approximately at 480 and 540 nm for the blue and green domains, respectively. The spectra did not appreciably change the shape due to the rotation of the analyzer (Fig. S2e). In both real (b) and reciprocal space (e), the polarization contrast (the ratio of maximal to minimal peak reflectance) was lower in the blue than in the green domains.

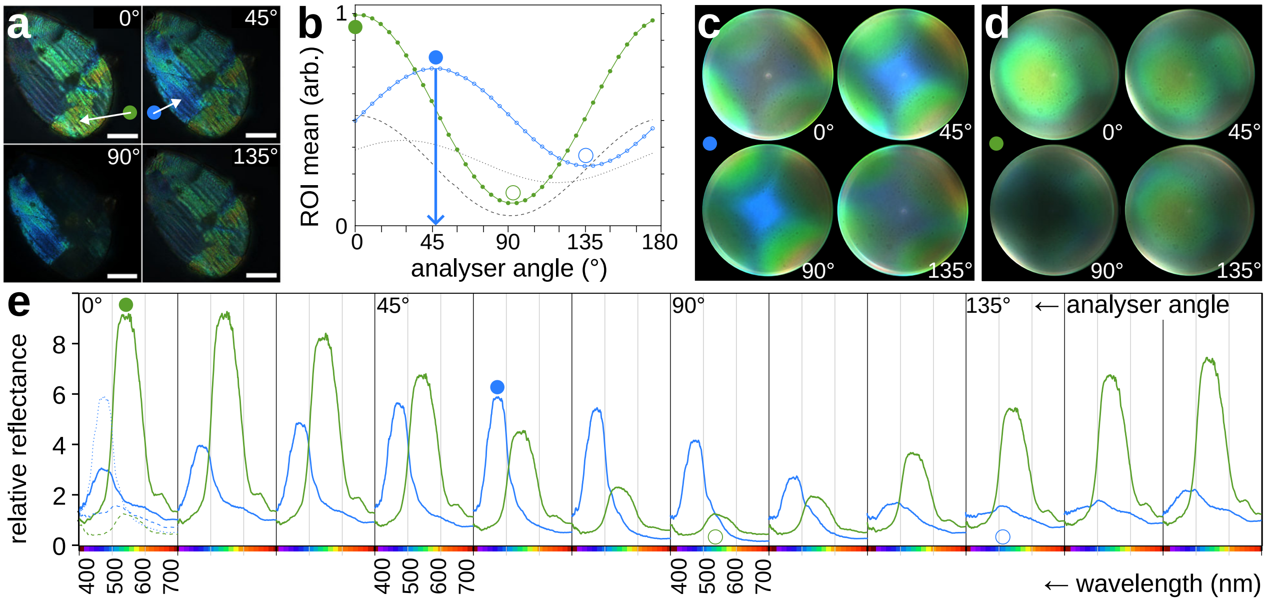


Figure S2: **Polarization properties of {100} and {111}- oriented domains.** These measurements were performed with a 100× (NA = 0.95) objective, a rotating analyzer and the polarizer fixed at 0°(vertical) orientation. **(a)** Real space images of a scale illuminated with a narrow aperture beam, with the analyzer set to 0°, 45°(*top row*), 90°, 135°(*bottom row*). **(b)** Polarization analysis of the image in (a). Average pixel values were extracted from RGB channels of the two ROIs indicated in (a). B channel of the blue domain (*blue solid line*); G channel of the green domain (*green solid line*); G/blue (*grey dotted line*), B/green (*grey dashed line*). **(c,d)** Reciprocal space images obtained from full-aperture illuminated spots, limited to blue and green domains. **(e)** Polarized reflectance spectra measured in reciprocal space close to the symmetry points X (*blue line*) and L (*green line*), referenced to a white diffuse standard. *Full and open dots* (b,e) indicate maximal and minimal reflectance amplitudes, respectively. *Scale bar* in (a,b): 10 μm. Image rim (c,d) corresponds to 71° (NA = 0.95).

The measured angle of rotation is dependent on the exact orientation of the domain. For the particular domain shown here, with an almost exact {100} orientation, the rotation was about 50°, measured

both in the center of the reciprocal space image, using full aperture illumination, and real space images using narrow aperture illumination. For arbitrary domain orientations close to the symmetry point X, the polarization rotation angle measured in real space images could be up to 90 °, while the domains orientated close to the symmetry point L produced rotations below 15 °(not shown).

## Estimation of the lattice parameter

We estimated the lattice parameter from the periodicity of the lattice in SEM images of plasma-etched scales (Fig. S3). The periodicity $k$ of the orientation $\{111\}$ was estimated as the average of the spatial frequencies of the three unique peaks from the DC point, and the nearest neighbor distance $2k/\sqrt{3}$ was multiplied with $\sqrt{2}$ to obtain the unit cell size.

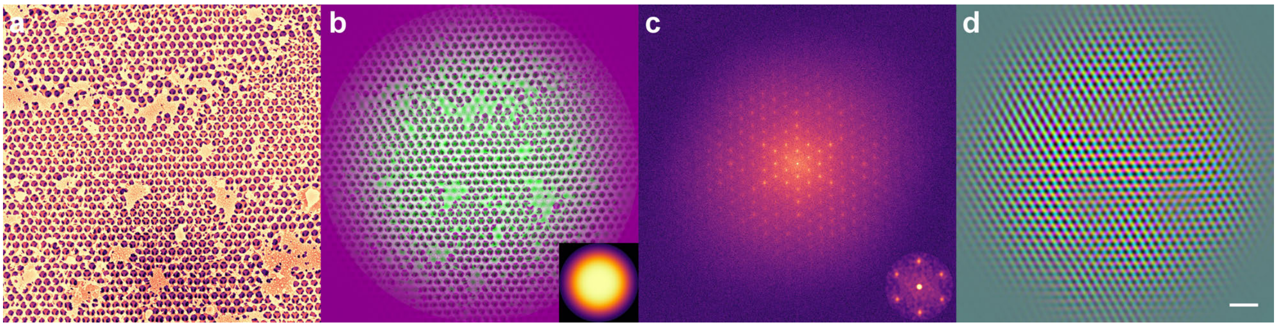


Figure S3: **Analysis of a hexagonal planar lattice corresponding to the $\{111\}$ orientation**. **(a)** A crop from the original SEM image. **(b)** A composite image of the windowed SEM image from (a) (*green channel*) and its low-pass filtered version (*magenta channel*). The low-pass filtered image was obtained from the k-space image (c) by selecting circular regions around the first six maxima. The window applied to the original image (a) before Fourier transform was a Gaussian-blurred circular mask (*inset*). **(c)** the k-space image obtained from the windowed SEM image shown in (b, *green channel*) using Fourier transform. The first six maxima (*inset*) are at a spatial frequency of about 270 nm per cycle, as measured with Fiji/ImageJ. **(d)** the lattice lines obtained via inverse transform of the k-space image with the opposite pairs of the lowest frequency peaks unmasked, mapped to the RGB channels. *Scale* for (a,b,d): 1 µm.

## Elemental composition of scales and titania replicas

We used the EDX detector (Oxford Instruments energy dispersive spectrometer, detector area 50 $mm^2$) installed on a Zeiss Ultra Plus scanning electron microscope to perform elemental analysis of native scales and the titania replica. The titanium fraction in the replicas (with gold coating contri-

bution discounted) was about 46% . The C:O:N ratio was (0.75 : 0.14 : 0.12) in the native scales and (0.35 : 0.65 : 0.00) in the replicas. The change of C:O ratio from 6.30 to 0.53 and disappearance of nitrogen indicated that the removal of the biological material during templating was satisfactory.

Table S1: Atomic percentage of the elements found in the original scales and negative titania replicas determined using EDX measurements.

| Element | Native scale [Atomic %] | TiO2 replica [Atomic %] |
|---|---|---|
| $C_K$ | 66.94 | 16.16 |
| $N_K$ | 12.67 | |
| $O_K$ | 10.63 | 30.74 |
| $Au_M$ | 8.51 | 15.32 |
| $Si_K$ | 1.25 | |
| $Ti_L$ | | 36.02 |

## Reflectance spectra of the replica with arbitrary lattice orientations

We have obtained several replicas with domain orientations away from {100} or {111}. For completeness, we report their reflectance spectra here (Fig. S4).

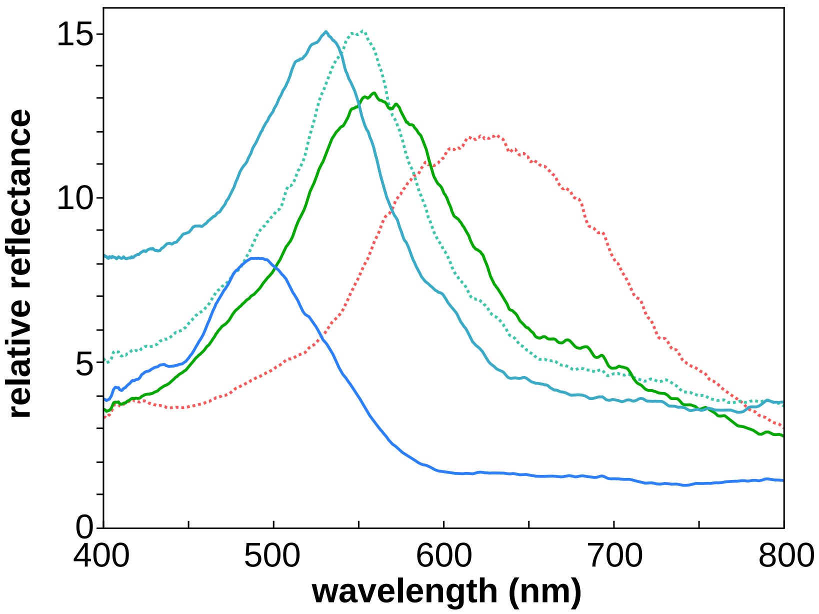


Figure S4: **Additional replica reflectance spectra.** The two spectra shown in the main text are shown with *dotted lines*. Diffuse white standard was used as a reference.

# Smooth transitions between crystallites

In native scales, the crystallites are continuous (Fig. S5. We observed both with light microscopy and SEM (not shown), that scales may also internally break, likely due to harsher hand manipulation. This often happens along the crystallite boundaries, indicating that crystallite lattice transitions may be mechanically weaker.

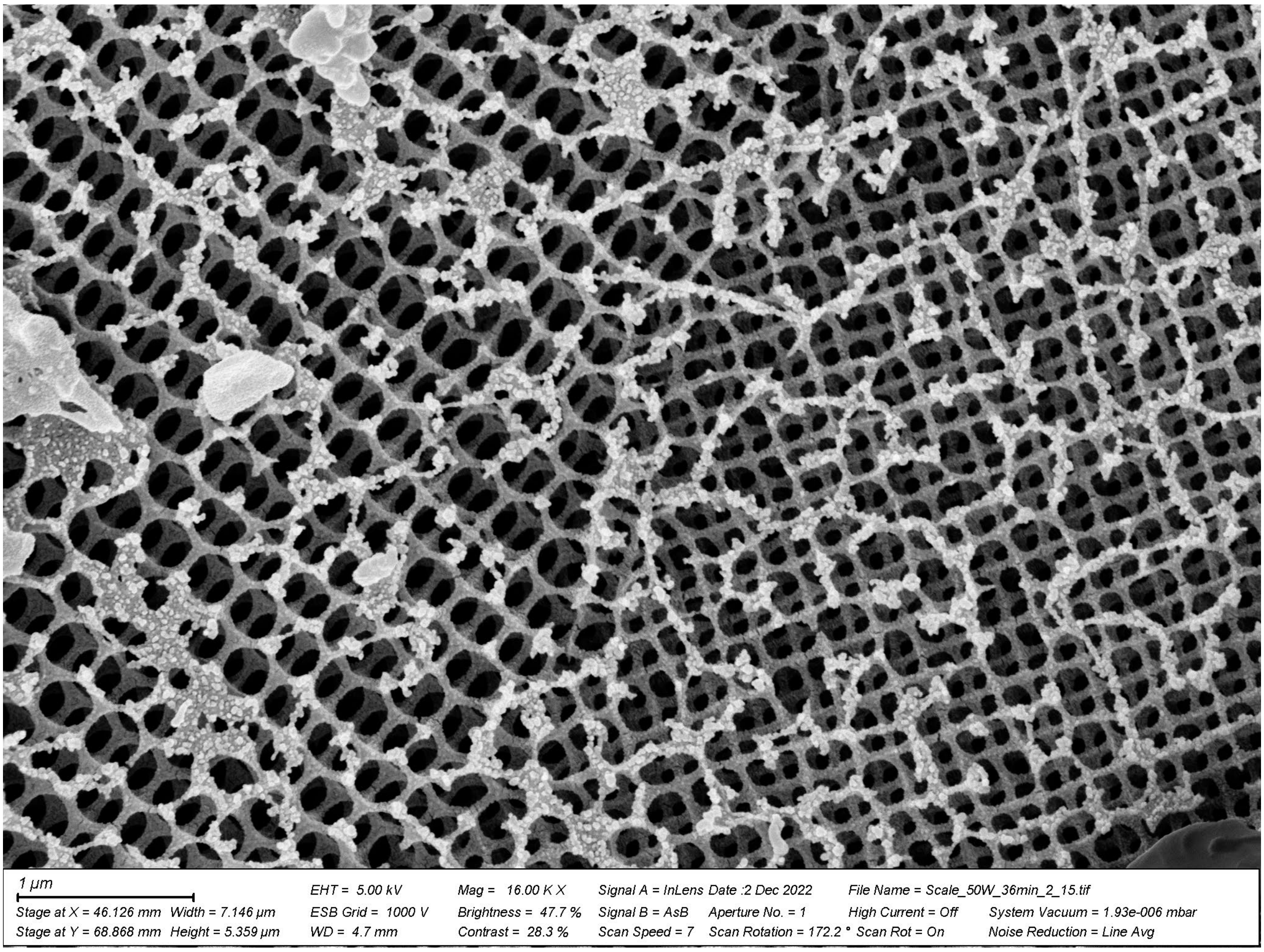


Figure S5: **Smooth transition between two lattice domains**. The two domains are in approximate orientations $\{111\}$ (*left*) and $\{100\}$ (*right*).

# Additional light microscopy and simulation of macroscopic color

The far fields taken under full aperture illumination (Fig. S6) were used for averaging (Fig. S7). The scales under the microscope look colorful and specular (Fig. S6, S7a), while the animal appears dull

under an extended light source (Fig. S7b). The effect of averaging was previously experimentally shown using a full hemisphere imaging scatterometer [3], see Figure 4 in [4]. The average of reciprocal space images taken with narrow full aperture illumination (Fig. S7d) simulates the situation when the weevil would be illuminated by diffuse sky, resulting in the diffuse appearance (Fig. S7b). The mechanism is discussed in [5]).

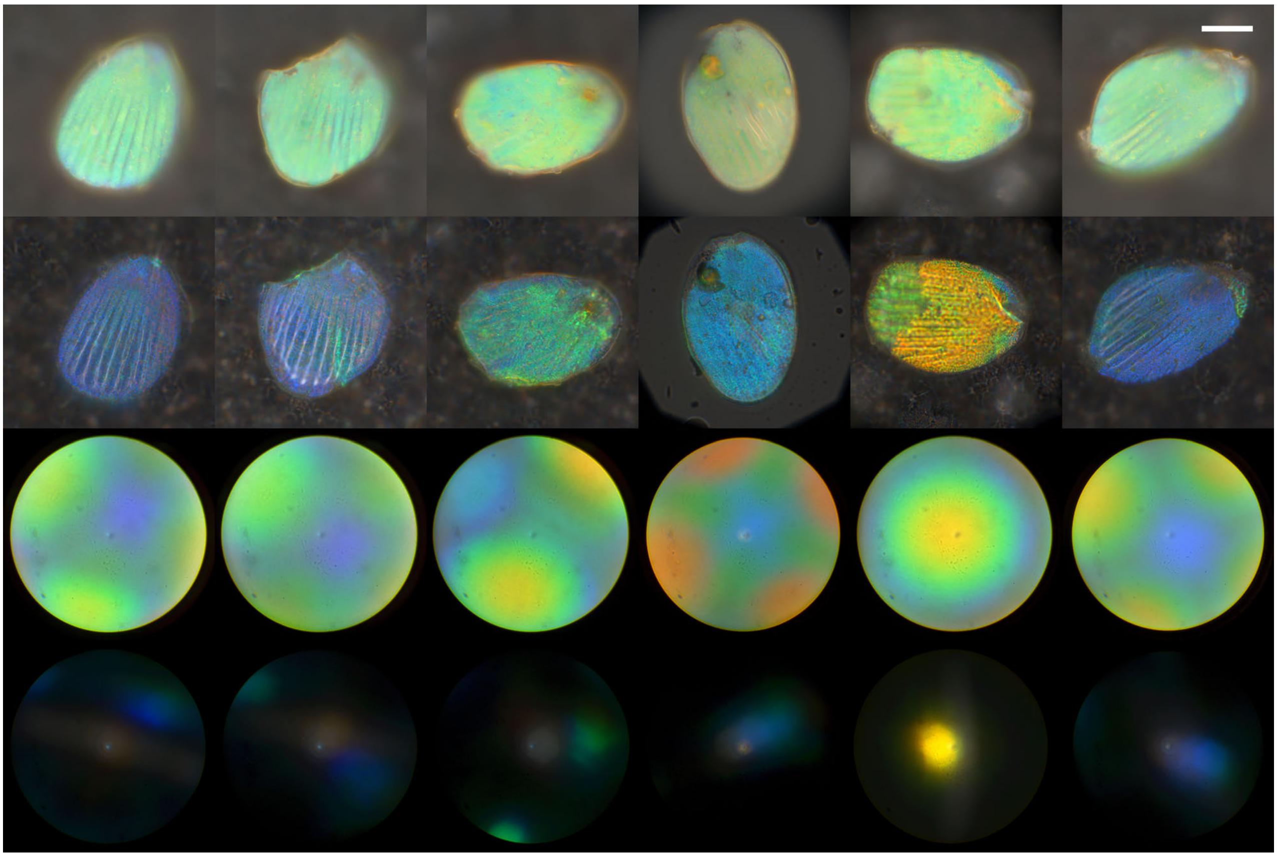

Figure S6: **Additional real and reciprocal space images of scales**. Real space images of the scales illuminated with open (*row 1*) and closed (*row 2*) aperture, and corresponding reciprocal space images (*row 3, 4*). The high symmetry points lie very approximately half way between the coloured speckles and the central spot artefact (*row 4*). Note that all scales but one (No.5) contain a single domain. *Scale bar* 20 µm. The rim of the reciprocal space images corresponds to NA 0.95.

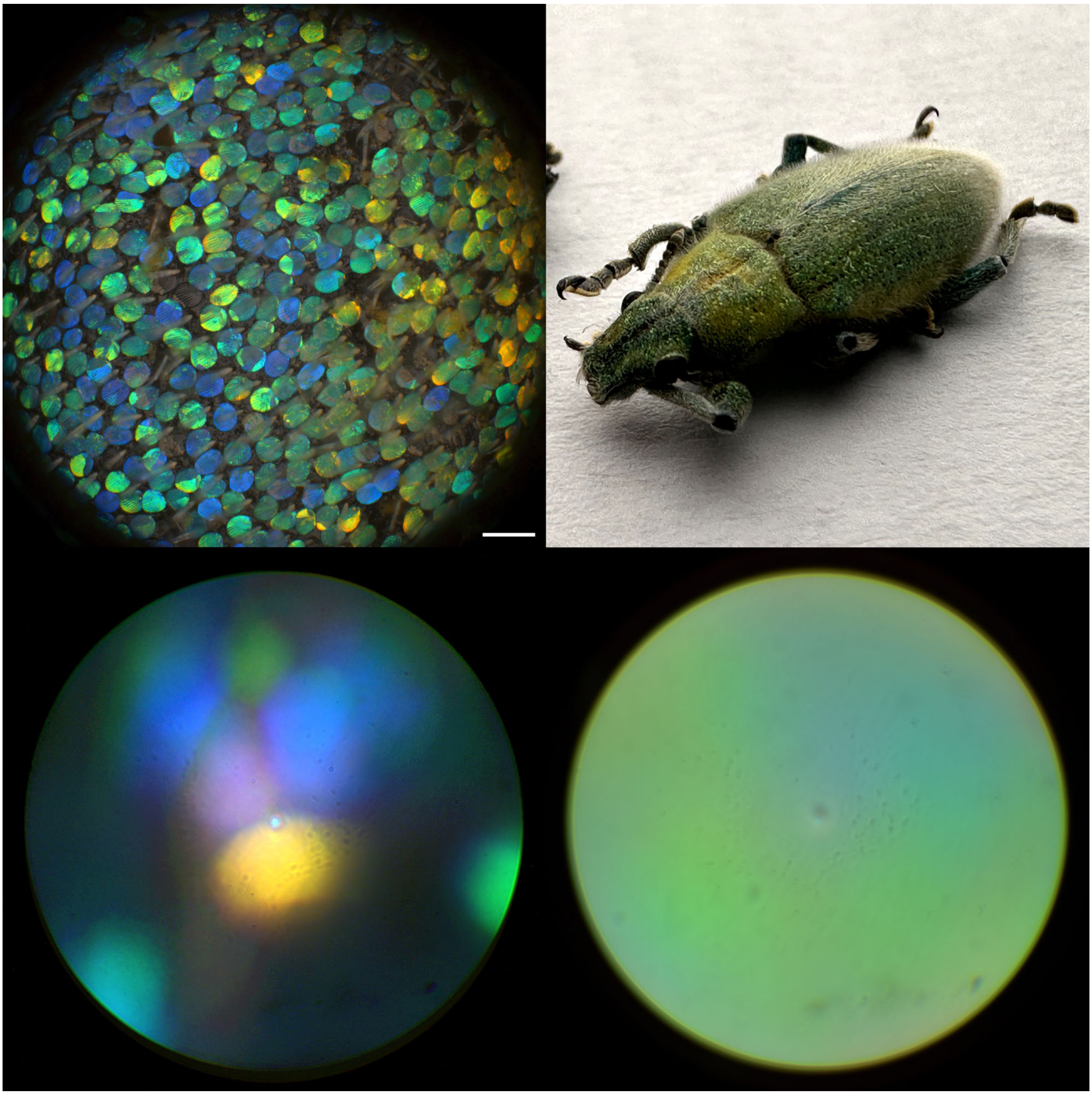

Figure S7: **Color production by averaging.** **(a)** *Top left*: The scale colors are randomly distributed, but the population of scales on the right side appears to have less blue and more yellow scales. These scales are observed under a slanted angle. Focus stack of images taken with a 20× (NA = 0.60) objective. **(b)** *Top right*: A photo of the animal taken under an extended source (60 × 60 cm LED panel), taken with an iPhone 14 Pro (Apple, Cupertino, CA, US). **(c)** *Bottom left*: A maximal projection of the six far field radiation patterns with the narrow aperture illumination. **(d)** *Bottom right*: Average of 14 far field images implies a uniform green spatial reflection. The rim of the reciprocal space images corresponds to NA 0.95. *Scale bar* (a) 0.1 mm

# References


1. Leertouwer, H. L.; Wilts, B. D.; Stavenga, D. G. Opt. Express **2011**, 19 (24), 24061–24066. doi:10.1364/OE.19.024061.

2. Wu, X.; Rodríguez-Gallegos, F. L.; Heep, M.-C.; Schwind, B.; Li, G.; Fabritius, H.-O.; von Freymann, G.; Förstner, J. Adv. Opt. Mater. **2018**, 6 (24), 1800635.

3. Stavenga, D.; Leertouwer, H.; Pirih, P.; Wehling, M. Optics Express **2008**, 17 (1), 193–202.

4. Wilts, B. D.; Michielsen, K.; Kuipers, J.; De Raedt, H.; Stavenga, D. G. Proceedings of the Royal Society B: Biological Sciences **2012**, 279 (1738), 2524–2530.

5. Seago, A. E.; Brady, P.; Vigneron, J.-P.; Schultz, T. D. Journal of the Royal Society Interface **2009**, 6 (suppl_2), S165–S184.
